# Porto-Sinusoidal Vascular Disorder: An Under-Recognized Liver Manifestation in Turner Syndrome

**DOI:** 10.3390/jcm14113979

**Published:** 2025-06-05

**Authors:** Sofia M. Siasiakou, Eleni Stoupi, Afroditi Roumpou, Amalia Papanikolopoulou, Nikolaos Syrigos, Dina Tiniakos, Melpomeni Peppa

**Affiliations:** 1 Third Department of Internal Medicine, Sotiria General Hospital for Chest Diseases, School of Medicine, National and Kapodistrian University of Athens, 11527 Athens, Greece; sofiasiasiakou@yahoo.com (S.M.S.); amaliapapaniko@yahoo.com (A.P.); nksyrigos@gmail.com (N.S.); 2 Department of Pathology, Aretaeion Hospital, School of Medicine, National and Kapodistrian University of Athens, 11528 Athens, Greece; eleni_st@windowslive.com (E.S.); dtiniak@med.uoa.gr (D.T.); 3 Endocrine Unit, Second Propaedeutic Department of Internal Medicine, Research Institute and Diabetes Center, Attikon University Hospital, School of Medicine, National and Kapodistrian University of Athens, 12462 Athens, Greece; 4 Translational and Clinical Research Institute, Faculty of Medical Sciences, Newcastle University, Newcastle upon Tyne NE17RU, UK

**Keywords:** Turner syndrome, porto-sinusoidal vascular disorder, obliterative portal venopathy, karyotype, aminotransferases, cirrhosis, steatosis, vascular liver disease, liver biopsy

## Abstract

**Background/Objectives:** Turner syndrome (TS) is a genetic chromosomal disorder including various manifestations depending on the karyotype; endocrine, gastrointestinal, respiratory, neurological, urogenital, musculoskeletal, and cardiovascular disorders contribute to increased morbidity and mortality. Liver function abnormalities are less well studied and mostly associated with insulin resistance, obesity, diabetes, hypogonadism, hypothyroidism, and autoimmune conditions. The association of liver pathology with architectural changes in various etiologies and the metabolic dysfunction-associated liver disease is of particular interest. **Methods**: Herein, we present three cases of adult women with TS and the persistent elevation of liver enzymes due to porto-sinusoidal vascular disorder (PSVD). **Results:** In one case, the diagnosis of TS followed the liver biopsy results. The absence of cardiometabolic risk factors, low liver stiffness and cardiovascular malformations may predict this histological diagnosis. **Conclusions**: Liver function impairment in TS may derive from a broad spectrum of liver pathology, including PSVD, and requires careful evaluation to decrease the risk of complications.

## 1. Introduction

Turner syndrome (TS) is a genetic chromosomal disorder of phenotypic females, consisting of one intact X chromosome and the complete or partial absence of the second sex chromosome. The mosaicism of the X chromosome (45,XO/46,XX or 45,X/47,XXX) or Y chromosome variants and X chromosome abnormalities (an isochromosome of either the p or q arm, the deletion of Xp or Xq, ring X, and mosaicism) may also be encountered. It affects about 1 in 2000 or 2500 live female births, but its true prevalence is unknown, as many individuals with mild phenotypes remain undiagnosed, while 99% of severely affected fetuses will not survive to term [1]. The median age at diagnosis is 15 years and is characterized by three age peaks: the prenatal period (during screening), between 5 and 20 years (due to short stature or delayed puberty), and between 30 and 40 years (due to infertility) [2]. TS presentation includes endocrine, gastrointestinal, respiratory, neurological, urogenital, musculoskeletal, and cardiovascular disorders that are associated with increased morbidity and mortality [3]. The severity of clinical presentation, morbidity, and mortality is proportional to the karyotype, with 45,XO being the most severe affected karyotype, followed by isochromosome X [1,4].

Liver function abnormalities are less well described in TS. They affect approximately 30–50% of patients, according to several population studies [5,6,7], and approximately 60–90%, according to matched control studies [8,9,10]. Several risk factors have been associated with liver disease, including isochromosome X karyotype, age, insulin resistance (IR), metabolic syndrome (MS), hypogonadism, hypothyroidism, autoimmune conditions, and cardiovascular disease [9,10]. Patients with TS have a 6-fold increased risk of cirrhosis compared to the general population [11], and liver disease seems to be the second most common cause of death in adult women with TS [12]. MS is associated with steatosis, with or without inflammation, which may progress to steatohepatitis with fibrosis, while CVD (cardiovascular disease) is associated with hepatic vascular architectural changes [13]. Recently, the latter has been included within the spectrum of porto-sinusoidal vascular disorder (PSVD), a group of hepatic vascular diseases in non-cirrhotic patients with lesions of portal vasculature and sinusoids, irrespective of the presence or absence of portal hypertension [14,15]. Liver biopsy is required for diagnosis, and the histological changes consist of portal and periportal vascular alterations, abnormal vascular relationships, and nodular regenerative hyperplasia (NRH). Conditions associated with PSVD include hematological diseases, prothrombotic conditions, immunological disorders, drug-induced liver injury, repeated gastrointestinal infections, and genetic diseases, including TS [15].

Herein, we present three cases of adult women with TS having a chronic and stable elevation of aminotransferases of unknown etiology that was finally attributed to PSVD. In addition, we discuss the possible pathogenetic associations and present a review of the literature.

## 2. Case Presentation

### 2.1. Case 1

A 22-year-old female has been followed at the hepatology unit due to elevated serum aminotransferases since the age of 16 years. Her medical history included a full-term 38-week labor, with normal birth weight, body length, and head circumference (3400 g, 51 cm, and 35 cm, respectively), astigmatism, hypermetropia, strabismus and iron deficiency anemia. Menarche occurred at 12 years old, with a normal menstrual cycle and complete pubertal development (Tanner stages: axillary hair AH = III, pubic hair PH = V, breasts B = V). She was only taking oral iron supplements and reported no allergy/smoking history or alcohol use. Phenotypically, she had short stature (a body height of 145 cm), a broad short-appearing neck, a low hairline at the back of the neck, deformity of the external ears, a broad chest, and a BMI (Body Mass Index) of 26 kg/m^2^. Clinical examination revealed neither specific (gastro-oesophageal or ectopic varices, portal hypertensive bleeding or porto-systemic collaterals) nor non-specific (ascites, platelet count <150,000 cells/μL or splenomegaly) signs of portal hypertension. Laboratory investigation, except for transient liver enzyme elevation, showed hypochromic microcytic anemia and mild thrombocytosis. Hepatitis virus screening, autoantibodies, serum γ-globulins, immunoglobulin G (IgG), α1-antitrypsin, copper, and ceruloplasmin were all negative (Table 1). The ultrasound scan of the heart, abdomen, and pelvis were normal. The Fibrosis 4 (FIB-4) score was 0.35, and the liver stiffness measurement was 6.9 kPa, indicating no significant fibrosis.

### 2.2. Case 2

A 30-year-old female with a known mosaic TS (45,XO/46,XX, 80% and 20%, respectively) diagnosed at the age of 9 months has been followed at the hepatology unit due to elevated liver enzymes for the past 4 years. Her medical history included a late-term 41-week labor, with a normal birth weight, body length, and head circumference (2600 g, 52 cm, and 35 cm, respectively). She was diagnosed with a bicuspid aortic valve and was operated on for coarctation of the aorta at the age of 9 months. Moreover, she underwent total thyroidectomy due to thyroid cancer at the age of 11 years and was diagnosed with astigmatism, hypermetropia, strabismus, and dyslipidemia. Menarche occurred at 17 years of age, with irregular menstrual cycles, menorrhagia, and incomplete pubertal development (Tanner stages: AH = II, PH = IV, B = III). She was treated with recombinant human growth hormone replacement therapy until puberty and took combined oral contraceptive pills for 10 years, until the age of 27. At present, she is treated with oral levothyroxine, hormone replacement therapy (HRT) (transdermal estradiol and oral progesterone), vitamin D supplements, and omega-3 triglycerides. She had no known allergies and no smoking history or alcohol use. Phenotypically, she had short stature (a body height of 153 cm), a short neck with a webbed appearance, a low hairline at the back of the neck, low-set ears, and a BMI of 17 kg/m^2^. Clinical examination revealed neither specific nor non-specific signs of portal hypertension. Laboratory investigation showed increased liver enzymes, whereas hepatitis virus screening, autoantibodies, serum γ-globulins, IgG, α1-antitrypsin, copper, and ceruloplasmin were all negative (Table 1). The liver ultrasound was normal. The FIB-4 score was 0.88, and the liver stiffness measurement was 2.6 kPa, indicating no significant fibrosis.

### 2.3. Case 3 

A 23-year-old female diagnosed with classic TS (45,XO) at the age of 6 years was followed at the endocrinology unit and was referred to the hepatology unit due to transiently elevated hepatic enzymes for the past 4 years. Her medical history included a full-term labor, with a normal birth weight, body length, and head circumference (2800 g, 50 cm, and 36 cm, respectively), a mild constriction of the aorta, chronic autoimmune thyroiditis, and an operated eardrum perforation, leaving hearing loss in her left ear. She was treated with human recombinant growth hormone replacement therapy until the age of 14 years, while puberty and menarche were induced with hormone therapy. At present, she is treated with oral levothyroxine, HRT (combined transdermal estrogen and progesterone), and vitamin D supplements. She had a normal menstrual cycle and complete pubertal development (AH = III, PH = V, B = V). She reported no known allergies, history of smoking, or alcohol use. Phenotypically, she had short stature (a body height of 150 cm), a short, webbed neck, a low hairline, ear deformity, a shield-shaped thorax, widely spaced nipples, and a normal BMI of 22.2 kg/m^2^. Clinical examination revealed neither specific nor non-specific signs of portal hypertension. Laboratory investigation did not indicate chronic liver disease (Table 1). The FIB-4 score was 0.57, the liver stiffness measurement was 4.5 kPa, and the liver ultrasound showed no abnormal findings.

### 2.4. Diagnostic Assessment 

**Figure 1:**
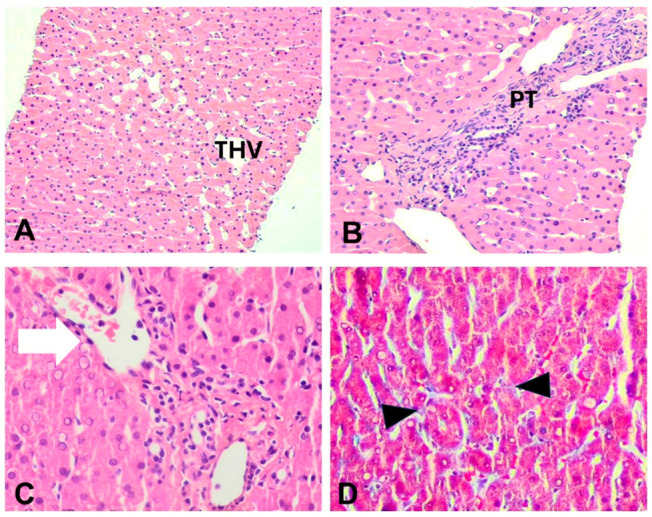
Liver histopathology of cases 1 and 3. (**A**) Case 3: zone 3 mild sinusoidal dilatation, H&E, ×200. (**B**) Case 3: portal tract with slit-like venule and abnormal periportal vessels, H&E, ×400. (**C**) Case 1: portal venule herniation (arrow), H&E, ×400. (**D**) Case 3: mild sinusoidal fibrosis (arrowheads), Masson trichrome, ×400. PT: portal tract, THV: terminal hepatic venule.

**Figure 2:**
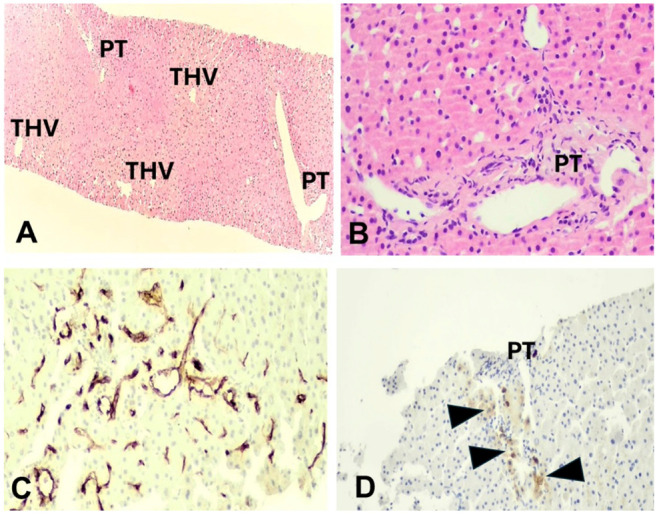
Case 2: (**A**) Sinusoidal dilatation and irregular liver vascular architecture, H&E ×40. (**B**) Hypovascularized portal tract, H&E, ×200. (**C**) Sinusoidal capillarization, immunostaining CD34, ×200. (**D**) Periportal intermediate keratin 7-positive hepatocytes (arrowheads) indicative of mild chronic cholestasis, keratin 7 immunostaining, ×100. PT: portal tract, THV: terminal hepatic venule.

A liver biopsy was performed in order to evaluate the underlying pathophysiology of persistent aminotransferase elevation. In all cases, an adequate sample was obtained, including 12–17 portal tracts. Parenchymal architecture was abnormal, with an irregular distribution of portal tracts, terminal hepatic venules, and mild non-zonal sinusoidal dilatation. On reticulin stain, areas of hepatocellular regeneration without nodule formation were detected. A few portal tracts were hypervascularized, containing a large number of thin-walled blood vessels. Focally, aberrant periportal thin-walled vessels were also noted. Rare portal venules were stenosed, suggestive of obliterative portal venopathy, while a few others were herniated, directly abutting into the neighboring liver parenchyma (Figure 1 and Figure 2). In cases 1 and 3, no portal inflammation or lesions indicative of cholangiopathy were noted. On the contrary, in case 2, there was mild portal chronic inflammation without interface activity and a mild loss of interlobular bile ducts (missing in 4/12 complete portal tracts), indicative of mild chronic cholangiopathy (Figure 2). Several periportal keratin 7 (K7)-positive intermediate hepatocytes were seen, indicative of mild chronic cholestasis (Figure 2D), while few centrilobular K7-positive hepatocytes were also present, indicative of mild ischemia. No significant lobular inflammation or steatosis was noted. In all cases, Masson trichrome staining highlighted mild sinusoidal and portal fibrosis without bridging septa, while CD34 immunostaining indicated focal sinusoidal capillarization in zones 2 and/or 3. The overall histology, as described above, was within the spectrum of PSVD, including one specific (obliterative portal venopathy/portal vein stenosis) and one or several non-specific histological features [14]. Of note, in case 1, the histological findings guided the genetic testing that showed karyotype monosomy 45, XO, consistent with TS.

## 3. Discussion

In contrast to other TS manifestations, liver disease is less well studied, and data regarding PSVD in TS are limited. Even though such cases have been described in the past as having NRH and/or obliterative portal venopathy [5,7,16,17], no large case series exist. We present three cases of adult women with TS and unexplained liver enzyme increase, all being at a low risk of developing advanced fibrosis and cirrhosis, according to FIB-4 and elastography measurements. On liver biopsy, overall histology was within the spectrum of PSVD. It is noteworthy that, in contrast to the other two cases, in case 1, PSVD diagnosis guided the diagnosis of TS. 

PSVD has recently been recognized as one of the disorders in the spectrum of liver disease associated with TS. It is a rare, novel, umbrella clinicopathological entity, encompassing vascular lesions affecting portal venules and sinusoids in the absence of cirrhosis. The term was introduced by the Vascular Liver Diseases Group to overcome limitations of the previously used “idiopathic non-cirrhotic portal hypertension” [14,15]. PSVD does not rely on the presence of portal hypertension and therefore includes asymptomatic patients at earlier stages of the disease. PSVD diagnosis requires an adequate liver biopsy excluding cirrhosis and one of the following: (1) the presence of either one specific histological sign or one specific sign of portal hypertension or (2) the combination of one non-specific histological sign and one non-specific sign of portal hypertension [14,15,18].

PSVD has been associated with a wide range of disorders, including autoimmune and hematological diseases, drug exposure, and various genetic syndromes, such as TS [15]. However, the exact pathogenetic mechanisms involved are not fully understood. It has been speculated that autoimmunity, vascular abnormalities, and thrombotic and possibly genetic predisposition may contribute to the development of PSVD [14,15,19].

Indeed, patients with TS face a 2–4 times increased risk of developing autoimmune disorders compared to the general population [20]. This has been attributed to the dysregulation of immunity tolerance and adaptation to antigen patterns due to the haploinsufficiency of X-linked genes [21]. In addition, TS patients are also at an increased risk of autoimmune conditions linked to hepatobiliary pathology, such as celiac disease and inflammatory bowel diseases (Crohn’s disease and ulcerative colitis), with a ranging prevalence of 0.67–4% [22]. Markedly, patients with PSVD without the syndrome may also have altered immunity; the increased activation of intrasinusoidal T lymphocytes may be responsible for damaging the endothelial cell lining of portal venules and sinusoids [23,24].

Liver parenchymal architectural changes in TS may be the consequence of primary vascular involvement [25]. Congenital cardiovascular malformations, including aortic coarctation (7–14%), bicuspid aortic valve (14–34%), and aortic dilation/aneurysm (3–42%), seem to progress rapidly [1,26]. Interestingly, in TS patients, aortic abnormalities may induce overt liver parenchymal architectural changes, including NRH, multiple focal nodular hyperplasia (FNH), and cirrhosis, that increase the risk of severe liver-related complications [25,26]. If a similar, rapid or slow, progression of vascular changes occurs in TS women, it could explain why liver involvement increases with age.

TS is also associated with venous thrombosis and an increased number of circulating prothrombotic agents, such as von Willebrand factor or factor VIII [27]. This prothrombotic state may lead to the repeated formation of micro-emboli inside the portal venule lumen, resulting in portal stenosis (obliterative portal venopathy) [27,28,29]. NRH is known to result from impaired hepatic blood flow and is a specific histological feature of PSVD [14].

TS-associated liver disease is often considered a consequence of metabolic and endocrine disorders, such as obesity and MS. IR, central obesity, diabetes, and hyperlipidemia are linked with a spectrum of liver injury, ranging from steatosis and steatohepatitis to cirrhosis [30]. Hypogonadism is frequently associated with metabolic dysfunction-associated liver disease (MASLD) [31]. Hypothyroidism may drive hepatic steatosis due to impaired lipid metabolism and hypothyroidism-induced myopathy [32].

Ultrasound is the most common technique for assessing liver and cardiac abnormalities in TS due to its accessibility, low cost, and safety. However, a significant number of structural abnormalities may not be diagnosed. Clinically, the differentiation of patients with PSVD exhibiting signs of portal hypertension from those with cirrhosis is challenging, often resulting in erroneous or postponed diagnoses [33]. Nonetheless, specific characteristics (e.g., reduced liver stiffness measurement, diminished hepatic venous pressure gradient, and increased spleen diameter in PSVD patients) may suggest a vascular origin of liver disease. Lampichler et al., in a retrospective study, used cross-sectional imaging (contrast-enhanced CT and/or dynamic MRI of the liver) to compare the radiological characteristics of PSVD with individuals who had cirrhosis or non-cirrhotic parenchymal liver disorders [34]. Several imaging features such as abnormal liver morphology, FNH-like lesions, and intrahepatic portal system abnormalities appeared to be associated with PSVD. In addition, periportal hyperintensity in the hepatobiliary phase of a gadoxetic acid-enhanced MRI was highlighted as a novel and specific characteristic in PSVD. A more recent systematic review including 12 studies [35] reported similar findings, with FNH-like lesions, intrahepatic and extrahepatic portal vein anomalies, and a larger spleen size favoring the diagnosis of PSVD, rather than surface nodularity and segment IV atrophy combined with segment I hypertrophy favoring the diagnosis of cirrhosis. For the identification and monitoring of thoracic aortic morphological abnormalities in individuals with TS, cardiovascular MRI is the gold standard because a transthoracic echocardiogram may underestimate the size of the aorta, the pericardial fluid, and the ventricular functional impairment and may not able to evaluate the anatomy in an irregular-shaped chest [36,37]. Modified versions of an active contour method have been proposed in order to increase the quantitative evaluation of cardiac function [38]. MRI angiography is also useful in identifying arterial and venous anomalies in TS patients [37]. 

Liver biopsy assessed by an expert hepatopathologist seems to be a useful diagnostic and probably prognostic tool in TS patients with persistently abnormal liver tests [39]. TS-associated liver injury presents with three main types of histology: (1) portal inflammatory infiltrate and steatohepatitis, (2) biliary lesions with periductal fibrosis, and (3) architectural changes. Roulot et al., in the largest clinicopathological study in TS [16], showed that patients without marked architectural changes do not experience liver-related complications and progressive liver disease. They proposed that cholangiopathy in TS patients without distorted liver tissue architecture may be related to altered blood supply and thus correspond to one end of a spectrum of vascular anomalies, with marked architectural changes being on the other end. If this hypothesis is correct, two distinct, but not exclusive, major histology patterns can be recognized: (1) steatosis and steatohepatitis, likely linked to overweight, IR, and MS, and (2) vascular abnormalities with architectural changes (with or without cholangiopathy), related to abnormal angiogenesis and vessel abnormalities (with or without autoimmune or thromboembolic disease). The first has low risk, whereas the second has an increased risk of liver-related complications. Nevertheless, recent studies have shown that PSVD without portal hypertension is characterized by an uncomplicated/benign course [40,41]. Bourcigaux et al., using both non-invasive tests and biopsy, reported that most patients (>90%) are at a low risk of developing fibrosis [7], although data suggest that TS women, in general, have a 6-fold increased risk of cirrhosis. 

In our series, none of our patients had obesity or MS. Two of them (cases 2 and 3) had constriction of the aorta. Liver biopsy revealed either no or mild inflammation. The overall histology was within the spectrum of PSVD. Roulot et al., in accordance with our findings, reported that architectural hepatic changes are the principal lesion in TS patients without obesity or metabolic dysfunction and might be part of a general vascular disorder [16]. In Calanchini’s study [5], half of the women with architectural hepatic changes had cardiac abnormalities and aortic dilatation. Recently, Bourcigaux et al. supported the link between NRH and a high aortic index [7]. In our study, patients with cardiovascular malformations (cases 2 and 3) presented liver enzyme elevations at 25 years and 21 years of age, respectively, suggesting a slow progression rate of liver histological changes. Interestingly, the mild chronic cholangiopathy in one case (case 2) coexisted with PSVD, whereas autoantibodies were all negative. This may indicate a relationship between chronic cholangiopathy and vascular abnormalities in TS.

According to a limited number of studies before 2019, approximately 20% of patients with obliterative portal venopathy have only liver biochemical abnormalities without any signs of portal hypertension at presentation [42]. Patients who presented with idiopathic noncirrhotic portal hypertension exhibited a mortality rate of 15–20% during a follow-up period of 8 years [43,44,45].

To date, data regarding the course of PSVD are rather sparse. However, according to two recent studies in adults, it seems that in the absence of portal hypertension, PSVD shows either slow or no disease progression [40,41]. The predisposing factors of portal hypertension in PSVD have not yet been identified; however it is thought that prothrombotic and immunological disorders are the most decisive ones [46].

Data regarding PSVD in TS are even more limited, and its course and prognosis are mostly unknown. Only a few cases have been described as having a poor clinical outcome, mainly due to complications of portal hypertension (variceal bleeding, ascites) [16,47].

Since liver biochemical abnormalities may be present in a significant subset of TS patients, a Doppler ultrasound is recommended to detect signs of portal hypertension and transient elastography to assess liver stiffness and evaluate the risk of advanced liver fibrosis and cirrhosis [25]. Liver biopsy may be considered after assessing the patient’s history, imaging studies, and clinical noninvasive fibrosis scores, such as FIB-4 [7]. HRT is essential for sexual development and metabolic and bone health in TS, but concern has been raised regarding the possible exacerbation of hepatic dysfunction. However, a trend towards a beneficial impact of HRT on TS-related liver disease has been observed in national patient registries [8], retrospective studies [48], and longitudinal studies [49]. Additionally, cardiometabolic risk factor management is of great significance in TS [1]. Improving metabolic parameters through lifestyle interventions (diet and physical activity), as well as maintaining a heathy body weight, not only reduces cardiovascular risk but also helps prevent liver disease [1,30,50].

## 4. Limitations of the Study

This is a series of three TS cases and the small sample warrants caution in the generalization of the results. More imaging with CT and MRI is missing, that could offer more data regarding specific liver and cardiovascular imaging features in TS patients with PSVD without cirrhosis. There is no long-term follow-up study of these patients due to the recent diagnosis. PSVD is a new entity that replaces and extends the previous term of idiopathic non-cirrhotic portal hypertension, including patients with only specific histological features without portal hypertension that were previously excluded from any disease entity. Therefore, there are no data of large cohorts regarding the incidence and the long-term outcomes of these patients. 

## 5. Conclusions

A multisystemic approach is required for TS patients with liver pathology. Screening for liver function must be included in the follow-up of people with TS. Although PSVD is rather uncommon, it is important to include it in the differential diagnosis of people with TS and persistently elevated liver enzymes, especially when they have cardiovascular malformations but no cardiometabolic risk factors. In this population, PSVD seems to be under-recognized and may be the consequence of primary vascular involvement.

## Figures and Tables

**Table 1:** Clinical and laboratory findings of the three patients.

	Case 1	Case 2	Case 3
**Age (years)**	22	30	23
**Labor (weeks)**	38	41	40
**Menarche** (years)	12	17	12 (induction)
**Puberty** (Tanner stages)	AH = III, PH = V, B = V	AH = II, PH = IV, B = III	AH = III, PH = V, B = V
**Body height** (cm)	145	153	150
**Medical history**	astigmatism hypermetropia strabismus iron deficiency anemia	astigmatism hypermetropia strabismus osteopenia dyslipidemia	constriction of aorta chronic autoimmune thyroiditis gonadal dysgenesis osteopenia insulin resistance hearing loss
**Karyotype test**	45,XO	45,XO/46,XX (fragment 80%/20%)	45,XO
**Phenotype characteristics**	short stature, broad short neck, low hairline, deformity of external ears, broad chest	short stature, short, webbed neck, low hairline, low-set ears	short stature, short, webbed neck, low hairline, ear deformity, shield-shaped thorax, widely spaced nipples
**Ht** (%)	38	38.6	39.4
**Hb** (g/dL)	11.5	12.8	13.7
**WBC** (cells/μL)	7620	6930	4630
**PLTs** (cells/μL)	528,000	190,000	189,000
**AST** (5–32U/L)	86	34	53
**ALT** (5–31 U/L)	106	37	130
**γ-GT** (5–36 U/L)	98	58	87
**ALP** (35–120 U/L)	125	149	71
**Total bilirubin** (mg/dL)	0.8	0.5	0.4
**Ferritin** (ng/mL)	13	120	32
**Glucose** (mg/dL)	81	92	93
**HBsAg**	(−)	(−)	(−)
**Anti-HBc**	(−)	(−)	(−)
**Anti-HBs**	(+)	(+)	(+)
**Anti-HCV**	(−)	(−)	(−)
**ANA**	(−)	(−)	(−)
**AMA**	(−)	(−)	(−)
**ASMA**	(−)	(−)	(−)
**IgG**	N	N	N

AH: axillary hair, PH: pubic hair, B: breasts, Ht: hematocrit, Hb: hemoglobin, WBC: white blood cell, PLTs: platelets, ALT: alanine aminotransferase, AST: aspartate aminotransferase, γ-GT: gamma-glutamyl transpeptidase, ALP: alkaline phosphatase, HBsAg: hepatitis B surface antigen, Anti-HBc: hepatitis B core antibody, anti-HBs: hepatitis B surface antibodies, anti-HCV: hepatitis C virus antibody, ANA: anti-nuclear antibody, AMA: anti-mitochondrial antibody, ASMA: anti-smooth muscle antibody, IgG: immunoglobulin G, N: normal, (+): positive, (−): negative.

## Data Availability

The original contributions presented in this study are included in the article. Further inquiries can be directed to the corresponding author.

## Abbreviations

ΒΜΙ: Body Mass Index, CVD: Cardiovascular Disease, FIB-4: Fibrosis 4 score, HRT: Hormone Replacement Therapy, IgG: Immunoglobulin G, IR: Insulin Resistance, MASLD: Metabolic Dysfunction-Associated Steatotic Liver Disease, MS: Metabolic Syndrome, NRH: Nodular Regenerative Hyperplasia, PSVD: Porto-Sinusoidal Vascular Disorder, TS: Turner Syndrome.
